# Letter to the editor: Triclosan in patients with inflammatory bowel disease

**DOI:** 10.4317/medoral.25407

**Published:** 2022-04-03

**Authors:** Juan Francisco Peña-Cardelles

**Affiliations:** 1 Head of Dentistry in Spanish Association of Crohn Disease and Ulcerative Colitis (ACCU); 2 Professor Oral Surgery. Department of Basic Health Sciences, Rey Juan Carlos University, Madrid, Spain; 3 Fellow Oral and Maxillofacial Surgery Department and Prosthodontics Department, School of Dental, University of Connecticut Health, Farmington, USA

In the Spanish Inflammatory Bowel Disease Society (ACCU) has been increasing the patient consults related to the possible relationship between triclosan in the development of acute ulcerative colitis pathology.

Even though triclosan is a molecule that has been used so far because of its great activity against dental caries, the FDA decided to ban it in different products in 2016 (1).

Ulcerative colitis is an autoimmune disease that, with Chron disease, are the group of the inflammatory bowel diseases (IBD). The prevalence of these diseases have been boosted during the last years in developed countries (2), and dentists have been updated regarding the management and risks in these patients, however, recent studies in mice have shown that triclosan is able to reach the intestinal mucosa and induces acute colitis in this animal model (3-5).

Due to the above-mentioned, there are patients concerned to use toothpaste or mouth rinses with this molecule among their ingredients. We have to clarify that the studies still are *in vivo* and invitro in model animals and not in humans, besides, this molecule is able to produce acute colitis in mice but it has not been studied in IBD mice. However, it could be prudent to use alternative products without triclosan to the oral hygiene of these patients until new studies show new outcomes.
